# Medical students’ knowledge and attitude toward brain death and the influence of medical education: a cross-sectional study

**DOI:** 10.1186/s12909-024-05346-w

**Published:** 2024-03-28

**Authors:** Chang Liu, Shiqing Liu

**Affiliations:** 1 Department of Psychology, Institution of Medical Humanities, China Medical University, Shengyang, China; 2 Department of Anesthesiology, Shengjing Hospital of China Medical University, No. 36 Sanhao Street, Heping Dist, 110004 Shenyang, China

**Keywords:** Brain death, Organ donation, Knowledge, Attitude, Medical student, China

## Abstract

**Background:**

The medical students’ knowledge and attitude toward brain death has not been investigated in China. The aims of this study were to assess the knowledge and attitude toward brain death among medical students in China and assess the influence of medical education on the knowledge and attitude.

**Methods:**

An online questionnaire consisting of 17 questions was developed and completed by undergraduates majoring in clinical medicine in China Medical University. The students’ demographic data, knowledge and attitude toward brain death were collected and analyzed.

**Results:**

A total of 1075 medical students participated in the survey, and 1051 of them completed the valid questionnaire. The exploratory factor analysis grouped the 17 items into four dimensions, which explained 63.5% of the total variance. These dimensions were named as knowledge (5 items), attitude (5 items), concern (3 items) and education needs (4 items) respectively. The global Cronbach α of the questionnaire was 0.845 and the Cronbach α of the four dimensions ranged from 0.756 to 0.866. The mean dimension scores of knowledge, attitude, concern and education needs was 3.67 ± 0.89, 3.67 ± 0.87, 3.10 ± 1.03 and 4.12 ± 0.72 respectively. The clinical students had a better knowledge than the preclinical students (*P* < 0.001). The clinical students had a more favorable attitude in stopping the treatment for a brain-dead family member and using the organs and/or tissues of brain-dead patients for transplantation (*P* < 0.001). The clinical students showed more concerns than the preclinical students (*P* < 0.001). There was no significant difference in the education needs between the clinical and pre-clinical students.

**Conclusions:**

Most medical students in China had insufficient knowledge about brain death. Although their knowledge of brain death increased with their university degree, their attitude toward organ donation after brain death did not evolve accordingly. Their concerns about brain death increased with seniority. Most students had great education needs about brain death.

**Supplementary Information:**

The online version contains supplementary material available at 10.1186/s12909-024-05346-w.

## Background

Organ shortage is a worldwide problem, and this problem is especially serious in China [1, 2]. The ratio of patients who are awaiting organ transplants to organ donors in China is about 150:1 while the number of organ donors per million population was only 2.0, which means that many people are dying on the waiting list [3]. Brain death is recognized by the law in most countries as the death of a person. However, there is no legislation on brain death in China. In west countries, most deceased donors were brain dead donors [4]. In China, however, nearly all of the deceased donors were from donation after cardiac death. Cultural and religious traditions have been referenced to explain that Asians are less likely to approve of brain death or organ transplant from brain dead donors [4, 5]. However, the lack of knowledge about brain death and organ donation among health professionals and the public in China might be another important factor [6, 7]. As the new generation of physicians, the medical students might be responsible for making the diagnosis of brain death and identifying potential organ donors in the near future. What’s more, their knowledge and attitude might influence the public’s attitude toward brain death. Although medical students’ knowledge and attitude toward brain death have been reported in many countries [8–13], there is limited evidence about the influence of medical education on the knowledge and attitude. The aim of this study was to investigate the knowledge and attitude toward brain death among medical students in China and to assess the influence of medical education on the knowledge and attitude.

## Methods

### Study design

The subjects of this study were undergraduates majoring in clinical medicine in China Medical University. Students in the first, second and third study year were classified as preclinical students and students in the fourth and fifth study year were classified as clinical students. This survey was performed using an online survey tool Questionnaire Star (Ranxing, Changsha, China) and the link to access the survey was distributed by the social software WeChat (Tencent, Shenzhen, China).

### Questionnaire

By reviewing previous publications focusing on the knowledge and attitude about brain death, 45 questions were extracted from these studies. After discussing with five experts (one neurosurgeon, one critical care medicine physician, one ethicist, one psychologist, and one organ transplant expert), 19 questions were used for the generation of the questionnaire. These questions can be divided into three categories, including knowledge, attitude and concerns about brain death. In addition, we also wanted to investigate the willingness of medical students to acquire and disseminate the knowledge about brain death. Therefore, we devised 4 questions related to this aspect. These 23 questions formed the initial version of questionnaire. To ensure the content validity, the initial version of the questionnaire was checked and modified by the same five experts.

This preliminary 23-item version of the questionnaire was administered to a sample of 200 medical students for pilot test in March 14, 2021. At the end of the questionnaire, students were asked to give their comments and suggestions on the questionnaire. After the pilot study, 6 questions were deleted with regard to redundancy or low response rates (missing data over 20%), and the final version of the questionnaire included 17 items (Appendix 1).

The survey started from March 29, 2021 and lasted two weeks. The final version of the questionnaire was shared by 5 coordinators in the Wechat group among the 5 grades respectively. To expand the sample size, the snowball sampling was adopted. The participants could share the link to the questionnaire in the WeChat group of their classes. In order to avoid repeated responses, each WeChat account was set to only fill in the questionnaire once. The students that had participated in the pilot test were excluded from the survey. The informed consent was obtained from each participant before the investigation. At the beginning of the survey, each participant was asked to fill in their basic demographic information, including age, gender, grade and religious beliefs. The participants in this study were anonymous, and they did not receive any compensation.

### Statistical analysis

Descriptive statistics were used to summarize the demographic data of the students. An exploratory factor analysis was conducted using principal component analysis with Varimax rotation to examine the factor structure of the 17 items of the questionnaire. The reliability of the questionnaire was measured by Cronbach alpha coefficient. The Pearson correlation was used to assess inter-dimension correlations. The chi-square test was used to compare the knowledge and attitude between pre-clinical students and clinical students. SPSS 23.0 (IBM Corp., Armonk, NY) was used for statistical analysis, and a p level of < 0.05 was considered statistically significant.

## Results

### Demographic data of study participants

A total of 1075 medical students participated in the survey, and 1051 of them completed the valid questionnaire. The number of students in grade one to grade five was 321, 163, 220, 113 and 234 respectively. Among these students, 407 (38.7%) were men and 644 (61.3%) were women. The mean age of the students was 20.6 ± 1.8 years. The demographic details of the students were shown in Table 1.

**Table 1:** Demographic data of the medical students

	Grade 1	Grade 2	Grade 3	Grade 4	Grade 5
	(*n* = 321)	(*n* = 163)	(*n* = 220)	(*n* = 113)	(*n* = 234)
Age (year)					
Mean ± SD	18.7 ± 0.8	19.9 ± 0.9	21.0 ± 0.8	21.8 ± 0.9	22.9 ± 1.0
Sex					
Male	132 (41.1%)	54 (33.1%)	100 (45.5%)	40 (35.4%)	81 (34.6%)
Female	189 (58.9%)	109 (66.9%)	120 (54.5%)	73 (64.6%)	153 (65.4%)
Religious belief					
Christianity	8 (2.5%)	3 (1.8%)	3 (1.4%)	1 (0.9%)	5 (2.1%)
Islam	0 (0.0%)	2 (1.2%)	1 (0.5%)	0 (0.0%)	0 (0.0%)
Buddhism	12 (3.7%)	4 (2.5%)	5 (2.3%)	5 (4.4%)	9 (3.8%)
No religion	301 (93.8%)	154 (94.5%)	211 (95.8%)	107 (94.7%)	220 (94.1%)

### Scoring

All items were measured on a 5-point Likert scale and the scores for item No.4 and No.5 were reversed so that higher scores indicated a higher level of knowledge. The score of each dimension for each student was obtained by averaging the item scores of the dimension.

### Validity and reliability of the questionnaire

The Kaiser-Meyer-Olkin sampling adequacy measure was 0.849 and the Bartlett’s test of sphericity was significant with a p value of < 0.001. The exploratory factor analysis grouped the 17 items into four dimensions, which explained 63.5% of the total variance (Table 2).

These dimensions were named as knowledge (5 items), attitude (5 items), concern (3 items) and education needs (4 items) respectively. The global Cronbach α of the questionnaire was 0.845 and the Cronbach α of the knowledge, attitude, concern and education needs were 0.756, 0.842, 0.792 and 0.866, respectively.

**Table 2:** Principal component analysis of the 17-item questionnaire

Item	Factors			
	Knowledge (11.5%)	Attitude (29.5%)	Concern (8.2%)	Education needs (14.3%)
1. Brain dead patients have no brainstem reflex.	0.684			
2. Brain dead patients do not breathe spontaneously.	0.725			
3. Brain dead patients have no awareness of their surroundings.	0.773			
4. Brain dead patients can feel pain.	0.627			
5. Brain dead patients may wake up.	0.652			
6. Stop the treatment for a brain dead family member.		0.587		
7. Organs and/or tissues of brain dead patients can be transplanted to the recipient.		0.780		
8. Donate my organs and/or tissues after brain death.		0.775		
9. Donate the organs and/or tissues of a brain dead family member.		0.784		
10. Accept organs and/or tissues donated by brain dead patients.		0.749		
11. Be misdiagnosed as brain dead and lose the chance of treatment.			0.841	
12. Be misdiagnosed as brain dead and organs and/or tissues might be harvested while alive.			0.833	
13. Premature termination of treatment.			0.774	
14. Participate in the training on the knowledge about brain death.				0.805
15. Added the knowledge about brain death to the curriculum of medical education.				0.836
16. Disseminate the knowledge of brain death to family members or friends.				0.830
17. Disseminate the knowledge of brain death to the public.				0.849

An exploratory factor analysis was conducted using principal component analysis with Varimax rotation to examine the factor structure of the questionnaire.

### Knowledge of and attitude toward brain death

#### Knowledge

The mean dimension scores of knowledge was 3.67 ± 0.89. Most students agreed that ‘Brain-dead patients have no brainstem reflex’ (58.1%), ‘Brain-dead patients do not breathe spontaneously’ (58.2%) and ‘Brain-dead patients have no awareness of their surroundings’ (67.7%). Most students disagreed that ‘Brain-dead patients can feel pain’ (60.5%) and ‘Brain-dead patients may wake up’ (56.6%). The clinical students had a higher dimension score (4.06 ± 0.88 vs 3.48 ± 0.93, p < 0.001) and their knowledge in these 5 questions were all better than the pre-clinical students (p < 0.001) (Table 3).

**Table 3:** A comparison of the knowledge and attitude toward brain death between the pre-clinical and clinical students using the chi-square test

	Pre-clinical students	Clinical students	p value
	(*n* = 704)	(*n* = 347)	
Brain-dead patients have no brainstem reflex			
Agree (*n* = 610)	350 (49.7%)	260 (74.9%)	< 0.001
Disagree/Unsure (*n* = 441)	354 (50.3%)	87 (25.1%)	
Brain-dead patients do not breathe spontaneously			
Agree (*n* = 611)	351 (49.9%)	260 (74.9%)	< 0.001
Disagree/Unsure (*n* = 440)	353 (50.1%)	87 (25.1%)	
Brain-dead patients have no awareness of their surroundings			
Agree (*n* = 712)	434 (61.6%)	278 (80.1%)	< 0.001
Disagree/Unsure (*n* = 339)	270 (38.4%)	69 (19.9%)	
Brain-dead patients can feel pain			
Disagree (*n* = 631)	380 (54%)	251 (72.3%)	< 0.001
Agree/Unsure (*n* = 420)	324 (46%)	96 (27.7%)	
Brain-dead patients may wake up			
Disagree (*n* = 595)	346 (49.1%)	249 (71.8%)	< 0.001
Agree/Unsure (*n* = 456)	358 (50.9%)	98 (28.2%)	
Stop the treatment for a brain-dead family member			
Agree (*n* = 589)	346 (49.1%)	243 (70.0%)	< 0.001
Disagree/Unsure (*n* = 462)	358 (50.9%)	104 (30.0%)	
Organs and/or tissues of brain-dead patients can be transplanted to the recipient			
Agree (*n* = 698)	436 (61.9%)	262 (75.5%)	< 0.001
Disagree/Unsure (*n* = 353)	268 (38.1%)	85 (24.5%)	
Donate my organs and/or tissues after brain death.			
Agree (*n* = 778)	510 (72.4%)	268 (77.2%)	0.096
Disagree/Unsure (*n* = 273)	194 (27.6%)	79 (22.8%)	
Donate the organs and/or tissues of a brain-dead family member.			
Agree (*n* = 489)	320 (45.5%)	169 (48.7%)	0.321
Disagree/Unsure (*n* = 562)	384 (54.5%)	178 (51.3%)	
Accept organs and/or tissues donated by brain-dead patients			
Agree (*n* = 775)	513 (72.9%)	262 (75.5%)	0.361
Disagree/Unsure (*n* = 276)	191 (21.1%)	85 (24.5%)	
Be misdiagnosed as brain dead and lose the chance of treatment			
Yes (*n* = 396)	226 (32.1%)	170 (49.0%)	< 0.001
No/Unsure(*n* = 655)	478 (67.9%)	177 (51.0%)	
Be misdiagnosed as brain dead and organs and/or tissues might be harvested while alive			
Yes (*n* = 425)	260 (36.9%)	165 (47.6%)	0.001
No/Unsure (*n* = 626)	444 (63.1%)	182 (52.4%)	
Premature termination of treatment			
Yes (*n* = 560)	344 (48.9%)	216 (62.2%)	< 0.001
No/Unsure (*n* = 491)	360 (51.1%)	131 (37.8%)	
Participate in the training on the knowledge about brain death			
Yes (*n* = 848)	578 (82.1%)	270 (77.8%)	0.097
No/Unsure (*n* = 203)	126 (17.9%)	77 (22.2%)	
Added the knowledge about brain death to the curriculum of medical education			
Yes (*n* = 888)	592 (84.1%)	296 (85.3%)	0.610
No/Unsure (*n* = 163)	112 (15.9%)	51 (14.7%)	
Disseminate the knowledge to family members or friends			
Yes (*n* = 890)	587 (83.4%)	303 (87.3%)	0.095
No/Unsure (*n* = 161)	117 (16.6%)	44 (12.7%)	
Disseminate the knowledge to the public			
Yes (*n* = 885)	689 (83.7%)	296 (85.3%)	0.494
No/Unsure (*n* = 166)	115 (16.3%)	51 (14.7%)	

#### Attitude

The mean dimension scores of attitude was 3.67 ± 0.87. Most students agreed to stop the treatment for a brain-dead family member (56.0%). Most students agreed that organs and/or tissues of brain-dead patients can be transplanted to the recipient (66.4%). Most students agreed to donate their own organs and/or tissues after brain death (74.0%), while only less than half of the students agreed to donate the organs and/or tissues of a brain-dead family member (46.5%). As for accepting organs and/or tissues donated by brain-dead patients, most students had a favorable attitude (73.7%). The clinical students had a higher dimension score (3.89 ± 0.83 vs. 3.56 ± 0.8, *p* < 0.001), more favorable attitude in stopping the treatment for a brain-dead family member and using the organs and/or tissues of brain-dead patients for transplantation (*p* < 0.001). There was no significant difference in the attitude toward donating one’s own organs/tissues (*p* = 0.096), donating the organs and/or tissues of a brain-dead family member (*p* = 0.321) and accepting organs and/or tissues donated by brain-dead patients (*p* = 0.361) between the clinical and pre-clinical students (Table 3).

#### Concern

The mean dimension scores of concern was 3.10 ± 1.03. In this dimension, 37.7% of the students were worried that they might be misdiagnosed as brain dead and lose the chance of treatment, 40.4% of the students were worried that they might be misdiagnosed as brain dead and their organs and/or tissues might be harvested while alive, and 53.3% of the students were worried that their treatment might be terminated prematurely if they had declared to donate organs after brain death. The clinical students had a higher dimension score (3.32 ± 1.08 vs. 2.99 ± 0.98, *p* < 0.001) and they showed more concerns in all of these 3 questions than the pre-clinical students (*p* < 0.001) (Table 3).

#### Education needs

The mean dimension scores of education was 4.12 ± 0.12. Most students agreed to participate in the training on the knowledge about brain death (80.7%) and add the knowledge about brain death to the curriculum of medical education (84.5%). Most students would like to disseminate the knowledge to family members or friends (84.7%) and the public (84.2%). There was no significant difference in the dimension score (4.11 ± 0.74 vs. 4.13 ± 0.70, *p* = 0.708) and all of these 4 questions between the clinical and pre-clinical students (Table 3).

#### The correlations between dimensions

The Pearson correlation analysis showed that the knowledge was positively related with the attitude (*r* = 0.413, *p* < 0.001), concern (*r* = 0.196, *p* < 0.001) and education needs (*r* = 0.173, *p* < 0.001), respectively.

## Discussion

Medical students’ knowledge of brain death had been reported in many studies [8–13]. Since different tools were used to evaluate medical students’ knowledge, it is difficult to directly compare the knowledge among different studies. However, all of those studies had shown that there was a lack of knowledge of brain death among medical students. In a study in United States, only 33% of all medical students had an expert level of understanding brain death [8]. In a multi-center study performed on 9598 medical students in Spain, only 67% of the respondents understood the brain death concept [12]. In our study, the percentage of correct responses for each question about brain death ranged from 50.6 to 66.7%, which also showed medial students’ knowledge of brain death was insufficient in China. Previous studies have shown that medical students’ knowledge can evolve over their university degree [8, 9, 12]. Similarly, in this study, students in the clinical years had better knowledge than students in the pre-clinical years. In China, although there is no specific brain death course in the medical curriculum, brain death-related knowledge is scattered in several different courses. The concept of brain death is first mentioned in the course of medical ethics in the third study year. The diagnosis of brain death and organ donation is taught in the course of critical care medicine and surgery in the fourth and fifth study year. Therefore, it is reasonable that students in the clinical years had a better knowledge of brain death.

Since the clinical students had a better knowledge, we had expected that they might also had a more favorable attitude toward brain death. However, there was only weak correlation between the knowledge and the attitude (*r* = 0.413). In this study, the clinical students were more favorable to stop the treatment for a brain-dead family member. This can be explained by that the clinical students were more likely to accept the concept of brain death, and further treatment would not bring benefits to their brain-dead family member. Although the clinical students were more in favor of using organs and/or tissues from brain-dead patients for transplantation, their attitudes toward donating or accepting organs and/or tissues after brain death were not more favorable than the pre-clinical students. This means that the attitude towards organ donation is not entirely determined by the knowledge. In this study, although most students would donate their own organs after brain death, less than half of the students would donate their family member’s organs. This means family factor might also be an important determinant. Although family overrule donation intentions is an issue in many countries [14–16], this issue is especially true in China. Without the permission of the family, it is almost impossible to donate the organs after death in China [17].

In this study, we also found that there were great concerns about brain death among medical students and the concerns increased over their university degree. Almost half of the clinical students were worried that they might be misdiagnosed as brain dead and more than half of them were worried that their treatment might be terminated prematurely if they had declared to donate organs after brain death. We think the worrisome of the medical students reflects their lack of knowledge about the diagnostic process of brain death and the operation of the organ donation system in China. However, medical education alone seemed to increase the worrisome. In fact, the brain death criteria in the Chinese guideline are among the strictest in the world [18]. However, since there is no legislation on brain death, any clinically declared brain death is actually ‘illegal’. Therefore, the diagnosis of brain death is rarely implemented in China. Since clinical students are more accustomed to cardiac death, they might not have enough knowledge in the diagnosis of brain death.

Similar with previous studies, most medical students in China had great education needs about brain death [9, 10, 12, 19]. What’s more surprising is that most medical students would like to disseminate the knowledge to their family members or friends and the public. Previous studies have showed that education can improve the attitude of medical students and physicians about brain death and organ donation [20, 21]. However, some studies have shown that there was only mild correlation between knowledge and attitude about brain-death organ donation [8, 12]. Therefore, we believe that it is necessary to integrate medical student education on brain death and organ donation. It should not only include medical knowledge, but also include law, ethics and other relevant contents. Since the attitude was also influenced by cultural and family factors [4, 5, 17], the education should be adapted to the cultural backgrounds and family discussions should be encouraged in the process of education about brain death [22, 23].

There are several limitations in this study. First, since this is an online survey, students who are interested in the topic about brain death might be more willing to participate in survey, which might lead to selection bias. Second, because this is a cross-sectional study, we can only assess the relationship between medical education and students’ knowledge and attitude toward brain death. Therefore, further prospective studies are needed to clarify the impact of education. Finally, this study was conducted at a single medical university in China, so the results from this study might not be generalized to medical students in other regions of China. Therefore, a multi-center study is needed to investigate medical students’ knowledge and attitude toward brain death in China.

## Conclusions

Most medical students had insufficient knowledge about brain death. Although their knowledge of brain death increased with their university degree, their attitude toward organ donation after brain death did not evolve accordingly. Their concerns about brain death increased with seniority. Most students had great education needs about brain death.

### Electronic supplementary material

Below is the link to the electronic supplementary material.

Supplementary Material 1

## Data Availability

The datasets used and/or analysed during the current study are available from the corresponding author on reasonable request.

## Abbreviations

BD: brain death
