# Supplementary material for: Medical students’ knowledge and attitude toward brain death and the influence of medical education: a cross-sectional study
Source: BMC Med Educ. 2024 Mar 28;24:346. doi: 10.1186/s12909-024-05346-w (PMC10979631; doi:10.1186/s12909-024-05346-w)
Supplement: Supplementary file 1 — Supplementary Material 1 [file 12909_2024_5346_MOESM1_ESM.docx]

Additional file 1. The ﬁnal version of the Knowledge of and Attitude Toward Xenotransplantation Questionnaire. Translated from Chinese to English. The questions were asked to the students in the Chinese language.

| ***To what degree do you agree that …*** |  |  |  |  |  |
| --- | --- | --- | --- | --- | --- |
| **1.** Brain-dead patients have no brainstem reflex. | 1.Strongly no | 2.No | 3.Unsure | 4.Yes | 5.Strongly yes |
| **2.** Brain-dead patients do not breathe spontaneously. | 1.Strongly no | 2.No | 3.Unsure | 4.Yes | 5.Strongly yes |
| **3.** Brain-dead patients have no awareness of their surroundings. | 1.Strongly no | 2.No | 3.Unsure | 4.Yes | 5.Strongly yes |
| **4.** Brain-dead patients can feel pain. | 1.Strongly no | 2.No | 3.Unsure | 4.Yes | 5.Strongly yes |
| **5.** Brain-dead patients may wake up. | 1.Strongly no | 2.No | 3.Unsure | 4.Yes | 5.Strongly yes |
| **6.** I will stop the treatment for a brain-dead family member. | 1.Strongly no | 2.No | 3.Unsure | 4.Yes | 5.Strongly yes |
| **7.** Organs and/or tissues of brain-dead patients can be transplanted to the recipient. | 1.Strongly no | 2.No | 3.Unsure | 4.Yes | 5.Strongly yes |
| **8.** I will donate my organs and/or tissues after brain death. | 1.Strongly no | 2.No | 3.Unsure | 4.Yes | 5.Strongly yes |
| **9.** I will donate the organs and/or tissues of a brain-dead family member. | 1.Strongly no | 2.No | 3.Unsure | 4.Yes | 5.Strongly yes |
| **10.** I will accept organs and/or tissues donated by brain-dead patients if needed. | 1.Strongly no | 2.No | 3.Unsure | 4.Yes | 5.Strongly yes |
| ***To what degree are you worried that …*** |  |  |  |  |  |
| **11.** I might be misdiagnosed as brain dead and lose the chance of treatment. | 1.Strongly no | 2.No | 3.Unsure | 4.Yes | 5.Strongly yes |
| **12.** I might be misdiagnosed as brain dead and my organs and/or tissues might be harvested while I am still alive | 1.Strongly no | 2.No | 3.Unsure | 4.Yes | 5.Strongly yes |
| **13.**My treatment may be terminated prematurely if I declared to donate organs after brain death | 1.Strongly no | 2.No | 3.Unsure | 4.Yes | 5.Strongly yes |
| ***To what degree would you be willing that …*** |  |  |  |  |  |
| **14.**I will participate in the training on the knowledge about brain death. | 1.Strongly no | 2.No | 3.Unsure | 4.Yes | 5.Strongly yes |
| **15.**Knowledge about brain death should be added to the curriculum of medical education. | 1.Strongly no | 2.No | 3.Unsure | 4.Yes | 5.Strongly yes |
| **16.**I will disseminate the knowledge to my family members or friends after I have gained the knowledge about brain death. | 1.Strongly no | 2.No | 3.Unsure | 4.Yes | 5.Strongly yes |
| **17.**I will disseminate the knowledge to the public after I have gained the knowledge about brain death. | 1.Strongly no | 2.No | 3.Unsure | 4.Yes | 5.Strongly yes |
